# Sustained Delivery System for Stem Cell-Derived Exosomes

**DOI:** 10.3389/fphar.2019.01368

**Published:** 2019-11-14

**Authors:** Andri K. Riau, Hon Shing Ong, Gary H. F. Yam, Jodhbir S. Mehta

**Affiliations:** ^1^Tissue Engineering and Stem Cell Group, Singapore Eye Research Institute, Singapore, Singapore; ^2^Corneal and External Eye Disease Department, Singapore National Eye Centre, Singapore, Singapore; ^3^Ophthalmology and Visual Sciences Academic Clinical Programme, Duke-NUS Medical School, Singapore, Singapore

**Keywords:** exosomes, sustained delivery, hydrogel, stem cell, biomaterials, regenerative medicine

## Abstract

Recent literature has ascribed that the paracrine action of stem cells is mediated by exosomes. Exosomes are nano-sized extracellular vesicles (30 to 100 nm) of endocytic origin that play important roles in intercellular communication. They have the ability to deliver various therapeutic effects, e.g., skin regeneration or cardiac function recovery, when applied topically or injected systemically. However, injection of exosomes has been shown to result in rapid clearance from blood circulation and accumulation of the exosomes in the liver, spleen, lung, and gastrointestinal tract can be found as early as 2 h after injection. Topical administration of exosomes on the skin or ocular surface would suffer the same fate due to rapid fluid turnover (sweat or tears). Biodegradable or highly porous hydrogels have been utilized to load exosomes and to deliver a sustained therapeutic effect. They can also prevent the exosomes from being cleared prematurely and allow the delivery of a more localized and concentrated exosome dosage by placing the hydrogel directly at or in the proximity of the target site. In this mini-review, we elaborate on the challenges of conventional exosome administration and highlight the solution to the shortcomings in the form of exosome-incorporated hydrogels. Different techniques to encapsulate exosomes and examples of hydrogels that have been used to create sustained delivery systems of exosomes are also discussed.

## Introduction

The discovery of the therapeutic potential of stem cells has been one of the most exciting advancements in the field of biomedicine (Trounson and McDonald, 2015). Decades of research in stem cell biology has significantly improved our understanding of mechanistic pathways that stem cells take in tissue repair and regeneration (Körbling and Estrov, 2003; Sanchez Alvarado and Yamanaka, 2014). Pluripotent and multipotent stem cells are known for their self-renewal capacity and ability to transform into multiple cell types. For this reason, they have the ability to replace tissue loss in degenerative conditions, injuries, or due to aging. Despite the overwhelming potential, there are drawbacks associated with the direct application of stem cells on the tissue damage site. Transplanted stem cells may undergo uncontrolled proliferation forming unwanted tissue mass resembling primitive tissue structures (Erdo et al., 2003; Volarevic et al., 2018). Tumorigenesis and mutagenesis have regularly been a safety issue to be taken into consideration in stem cell therapy (Cairns, 2002; Erdo et al., 2003; Volarevic et al., 2018). Reports have also shown that many pluripotent stem cells, including induced pluripotent stem cells (iPSCs), are able to induce teratoma formation in a much faster rate than embryonic stem cells. This further raises the question regarding the safety of stem cell therapies (Gutierrez-Aranda et al., 2010; Zhang et al., 2011). Another common complication of allogeneic stem cell transplantation is graft-versus-host disease (GVHD). Although the risk can be minimized by donor-recipient matching, patients are still required to undergo long-term administration of immunosuppressive drugs. There is also a possibility of infection of these cells by contaminating bacteria, viruses or fungi that can transmit diseases to the recipients, particularly to patients receiving hematopoietic stem cell transplantation (Marr et al., 2004; Pascutti et al., 2016; Cho et al., 2018). In addition, improper handling methods, storage, and transportation can be detrimental to stem cell quality; potentially affecting the success rate of the treatment (Herberts et al., 2011).

There is substantial evidence that stem cells exert their therapeutic effect *via* secretion of soluble factors, as well as the production of exosomes (Lai et al., 2010; Raposo and Stoorvogel, 2013; Lin and Du, 2018). Exosomes are nano-sized vesicles (30 to 100 nm) of endocytic origin that play a pivotal role in intercellular communication (Raposo and Stoorvogel, 2013). The exosomes are released by every cell into extracellular environment. Their therapeutic effect takes place when they are internalized or in some cases, attached on the cell surface, and the effect typically depends on the content they carry, which includes DNA, proteins, mRNA, lipids, and miRNA (Raposo and Stoorvogel, 2013; Colombo et al., 2014). The content may vary depending on the physiological and pathological state of the cells from which the exosomes originate (Colombo et al., 2014; Schorey and Harding, 2016).

The use of exosomes in patients has several potential advantages: (i) Their use avoids the transfer of cells, which may have immunogenic molecules and even mutated or damaged DNA. The cell-free nature of exosomes makes it more favorable to regulatory bodies; (ii) The exosomes are small and can readily circulate through any organ, whereas cells are too large to circulate easily through capillaries and many do not get beyond the first pass capillary bed (Verweij et al., 2019); (iii) As exosomes are native to the body, their surface has inherent biochemical properties that are similar to cells, hence, they are able to avoid phagocytosis, fuse with cell membranes, and also bypass lysosomal engulfment (Xu et al., 2018a). The fact that exosomes are a natural product of the body results in a low immune response (EL Andaloussi et al., 2013); (iv) Exosomes have unique homing characteristic due to unique membrane proteins and lipids that bind to specific receptors on the recipient cell surface (Quah and O’Neill, 2005). However, delivering a therapeutic dosage of exosomes to the target cells, particularly *via* systemic injection, is not always as straightforward as it seems and has its challenges. This mini-review highlights those challenges and the solution to the shortcomings in the form of exosome encapsulation in biodegradable or highly porous hydrogels. Strategies to encapsulate soft nanoparticles, such as exosomes, and examples of materials that have been used for sustained delivery of stem cell-derived exosomes are also discussed.

## Challenges in Exosome Delivery

The intended biological effects of exosomes can only be produced as a result of internalization by target cells *via* an endocytic pathway (Mulcahy et al., 2014). The ability to prolong the half-life of exosomes at the target site is crucial in order to achieve the therapeutic dosage of the exosomes. Studies have shown that direct intravenous, intraperitoneal or subcutaneous injection of exosomes results in rapid clearance from the blood circulation and accumulation in the liver, spleen, lung, and gastrointestinal tract (Takahashi et al., 2013; Smyth et al., 2015). Regardless of the delivery route and cell source, the majority of systemically injected exosomes are rapidly taken up by macrophages in the reticuloendothelial system to be ejected from the body (Wiklander et al., 2015; Charoenviriyakul et al., 2017). The half-life of topically applied exosomes, e.g., on skin or ocular surface, may even be shorter due to the rapid clearance of vesicles by fluid (sweat or tears) and exposure to external elements. Topical application of drugs on the ocular surface has always resulted in low bioavailability due to the presence of epithelial tight junctions and rapid tear turnover (Agrahari et al., 2016).

Another issue that further advocates the need for a sustained delivery system of exosomes is the difficulty in producing the vesicles not only in a large quantity, but also in high purity and consistent quality (Yamashita et al., 2018). The large scale production for clinical studies and commercialization can become expensive (Taylor and Shah, 2015). The typical yield of an exosome isolation can be less than 1 µg of exosomal protein from 1 ml of culture medium (Yamashita et al., 2016; Charoenviriyakul et al., 2017), whereas the therapeutic dose of exosomes is usually in the range of 10–100 µg of protein in mouse model (Willis et al., 2017). In humans, the effective dose could be an order of magnitude or more to compensate for the rapid clearance of exosomes from the body. Biodegradable or porous hydrogels can be used to load a relatively low amount of exosomes, but still be able to produce the intended therapeutic effect and sustain the effect over a period of time, because hydrogels can prevent the encapsulated exosomes from being cleared prematurely (Liu et al., 2018). In addition, they also allow the delivery of a more localized and concentrated dosage by placing the exosome-loaded hydrogel directly at or in the proximity of the target site.

## Exosome Encapsulation Strategies

Hydrogels have been extensively used to create drug delivery systems with desirable therapeutic effects (Caló and Khutoryanskiy, 2015). They are crosslinked, three-dimensional hydrophilic polymer networks that form matrices with high water content (Peppas et al., 2006). The polymers commonly used to prepare the hydrogels are from natural (e.g., collagen, gelatin, chitosan, hyaluronic acid or alginate) or synthetic (e.g., poly(ethylene glycol) (PEG), poly(lactic-co-glycolic acid) (PLGA) or poly(hydroxyethyl methacrylate (pHEMA)) origins or the combination of both (Peppas et al., 2006). Hydrogels typically have tunable physical properties that can be taken advantage of to customize the degradation rate of the matrices to release the entrapped exosomes. They also have similarities to the native extracellular matrix (ECM), excellent biocompatibility, and malleable (Annabi et al., 2014). With these combined characteristics, hydrogels are an excellent candidate to encapsulate exosomes.

**Figure 1:**
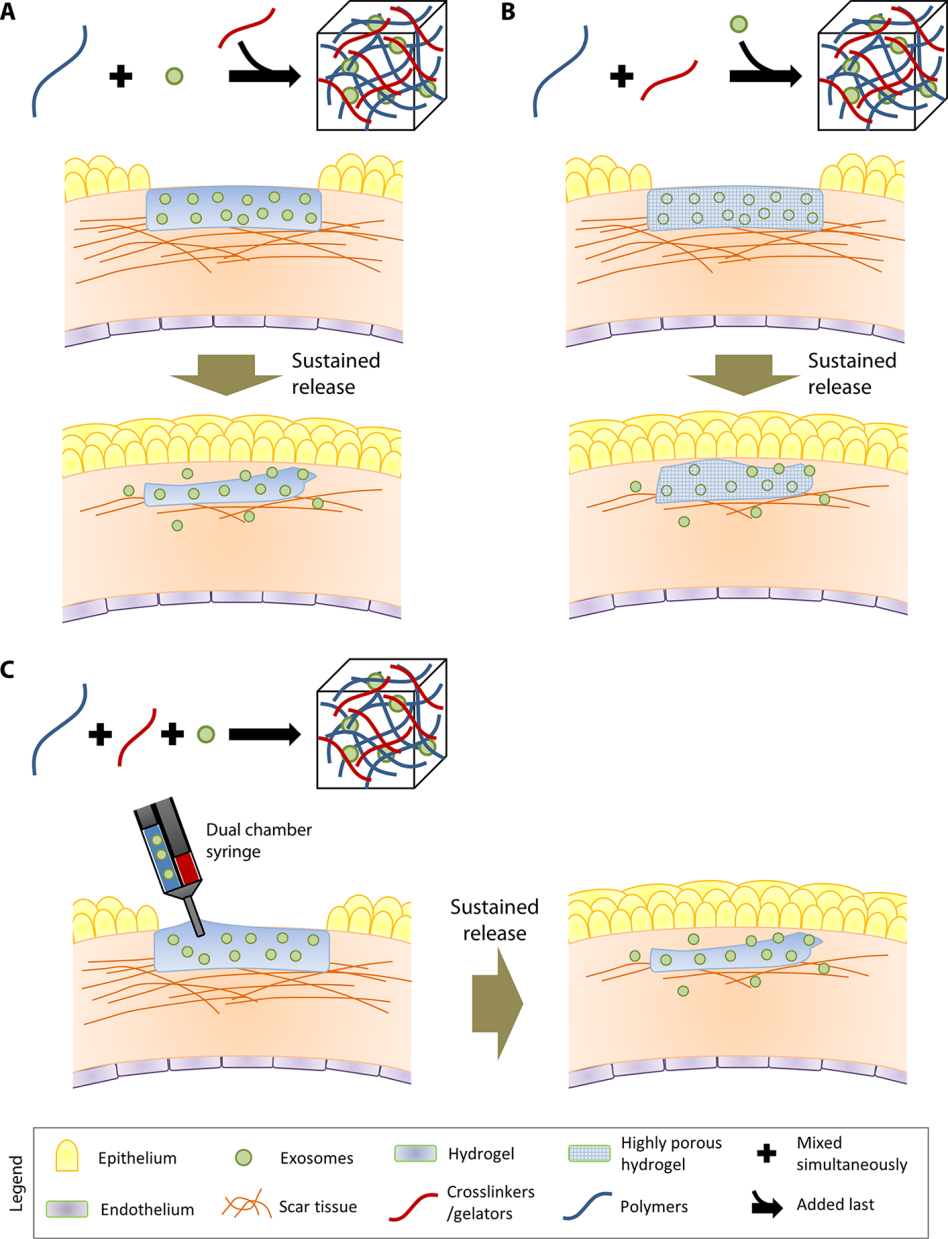
Three different methods to incorporate exosomes in hydrogels. In the illustrations, the therapeutic application of the exosome-loaded hydrogels is exemplified by their role in reducing scar tissue in the cornea. **(A)** The first strategy involves incorporation of exosomes by mixing with the polymers, followed by addition of crosslinkers to gel the composite. **(B)** The second strategy involves physically incorporation of exosomes after the gelation of the hydrogel. **(C)** The third strategy involves mixing the polymers, exosomes, and crosslinkers simultaneously. In situ gelation can be achieved by injecting the three hydrogel components using a dual-chamber syringe directly at the target site.

Encapsulation of exosomes into the hydrogel matrix can be performed in three ways:

1. Exosomes can be incorporated by mixing with the polymers, followed by addition of crosslinkers to gel the composite (**Figure 1A**). An example of this method would be the composite hydrogel created by Qin and coworkers (Qin et al., 2016). They utilized HyStem^®^-HP hydrogel, which is a composite substrate containing thiolated hyaluronic acid, thiolated heparin and thiolated gelatin (Ghosh et al., 2005), to incorporate bone marrow stem cell (BMSC)-derived exosomes. The thiolated polymers and exosomes were crosslinked/gelated with the addition of poly(ethylene glycol) diacrylate (PEGDA).
2. Exosomes can also be physically incorporated after the polymerization of hydrogel (**Figure 1B**). This incorporation technique is sometimes known as “breathing” method (Thomas et al., 2009). The “breathing” typically consists of placing the swollen hydrogel into a solvent to remove the entrapped water, followed by soaking the hydrogel in an aqueous solution containing the exosomes that causes the hydrogel to swell and “breath in” the exosomes. The technique requires hydrogels with pores larger than the exosomes, such as the chitosan/silk fibroin hydrogel sponge that was used by Xu and coworkers to encapsulate platelet-rich plasma exosomes (Xu et al., 2018b). The exosomes that are weakly attached to the matrices would be able to leach through the large pores.
3. It is possible to incorporate exosomes by mixing them with the polymers in solution form and crosslinkers simultaneously (**Figure 1C**). This technique would enable an *in situ* gelation, allowing direct injection of the hydrogel components (exosomes + polymers in solultion form + crosslinkers) at the target site. An example of the technique was carried out by Wang and colleagues, where they encapsulated adipose-derived mesenchymal stem cells (MSCs) in a polypeptide hydrogel made of Pluronic F127, oxidative hyaluronic acid, and poly-ε-L-lysine (Wang et al., 2019).

## Sustained Delivery System for Stem Cell-Derived Exosomes

Research into encapsulating exosomes in a hydrogel is still in its infancy; hence, there are currently only a limited number of studies in the literature. **Table 1** summarizes the studies that have described the use of hydrogel to encapsulate exosomes and the duration of release achieved by each type of material.

**Table 1:** Materials used to encapsulate exosomes derived from various cell sources.

Materials	Cell source	Duration of release	Clinical application of delivery system	Reference
Adamantane and β-cyclodextrin-modified hyaluronic acid hydrogel	Bone marrow-derived endothelial progenitor cells	21 days	Cardiac regeneration in infarcted heart	Chen et al. (2018)
Alginate hydrogel	Blood plasma	4 days	Skin regeneration in chronic diabetic wound	Guo et al. (2017)
Collagen type I Gelfoam^®^ sponge	Cardiomyocyte-derived IPSCs	21 days	Cardiac regeneration in infarcted heart	Liu et al. (2018)
Chitosan hydrogel	miR-125-3p-overexpressing synovium MSCs	6 days	Skin regeneration in chronic diabetic wound	Tao et al. (2017)
Chitosan hydrogel	Placenta MSCs	Not reported	Angiogenesis promotion in ischemic tissue	Zhang et al. (2018)
Chitosan/silk fibroin sponge	Blood plasma	Not reported	Skin regeneration in chronic diabetic wound	Xu et al. (2018b)
HyStem^®^-HP hydrogel	BMSCs	Not reported	Bone regeneration	Qin et al. (2016)
pH-responsive polypeptide (Pluronic F127, oxidative hyaluronic acid and poly-ε-L-lysine) hydrogel	Adipose MSCs	21 days	Skin regeneration in chronic diabetic wound	Wang et al. (2019)
Self-assembled peptide amphiphile (C16- GTAGLIGQ-GG-GHRPS) hydrogel	Umbilical cord MSCs	21 days	Cardiac regeneration in infarcted heart	Han et al. (2019)

Qin et al. were the first to describe the idea of encapsulation of exosomes in a hydrogel (Qin et al., 2016). In an effort to stimulate skin regeneration in diabetic rats with chronic skin wounds, Guo and colleagues loaded exosomes isolated from platelet-rich plasma (PRP) in sodium alginate hydrogel and observed a 96-h exosome release (Guo et al., 2017). In a separate study by Tao et al., also with the intention to accelerate skin wound healing, they loaded exosomes derived from miR-125-3p-overexpressing synovium MSCs in chitosan hydrogel (Tao et al., 2017). Patching of the exosome-loaded hydrogel over the skin wound resulted in significantly more rapid healing and more new vessel formation compared to controls (untreated skin and skin treated with the blank hydrogel). Entrapping platelet-rich plasma exosomes in chitosan/silk sponge resulted in close to 20% faster skin wound healing than untreated skin wound of diabetic rats after 15 days (Xu et al., 2018b).

The above studies have only shown a rather short particle release period (under 1 week). For certain clinical applications, such as treatment of myocardial infarction, the ability to deliver the exosomes for a longer period of time might be more practical in order to avoid repeated implantation of newly loaded hydrogel within a short period of time in target sites that are challenging to access. By encapsulating exosomes in collagen type I Gelfoam^®^ mesh, Liu et al. observed a 21-day release of exosomes isolated from cardiomyocyte-derived IPSCs (Liu et al., 2018). The cardiomyocyte-derived IPSCs were shown to be enriched in miRNAs that were beneficial in reducing infarct size, hypertrophy, and apoptosis in a rat model of acute heart infarction. Chen and et al. loaded exosomes isolated from bone marrow-derived endothelial progenitor cells in an injectable hyaluronic acid hydrogel that was modified with adamantane and β-cyclodextrin, and observe a linear particle release profile over 21 days (Chen et al., 2018). The exosome-loaded hydrogel resulted in better recovery of cardiac functions at 4 weeks after the onset of myocardial infarction *in vivo*, compared to the rats treated with free exosomes. By adding β-glycerophosphate in chitosan solution, it enabled *in situ* gelation of chitosan hydrogel loaded with placenta MSC-derived exosomes (Zhang et al., 2018). Although the release duration was not reported, by extrapolating the number of particles released per hour, the duration of exosome release from the injectable chitosan hydrogel was approximately 16 days. In their rat model of hindlimb ischemic injury, exosome-loaded hydrogel induced less fibrotic and necrotic tissue formation, inflammatory response, and hence, faster physiological function recovery compared to rats treated with free exosomes (Zhang et al., 2018).

A more complex hydrogel system capable of delivering exosomes and antimicrobial effect was introduced by Wang et al. (2019). The hydrogel, composed of pluronic F127, hyaluronic acid, and poly-ε-L-lysine, is pH-sensitive, where the adipose-derived MSC exosome release rate was more rapid in acidic pH than in neutral pH. The skin regeneration over the wound, injected with exosome-loaded hydrogel, was more rapid than that injected with free exosomes over 21 days. Han et al. introduced a complex injectable peptide amphiphile (PA) that could self-assemble into a hydrogel (Han et al., 2019). The authors incorporated umbilical cord MSC-derived exosomes into a PA with a 16-carbon-alkyl tail that was functionalized with cardioprotective peptide GHRPS (His-D-2-methyl-Trp-Ala-Trp-D-Phe-Lys) and matrix metalloprotease-2 (MMP-2) degradable sequence GTAGLIGQ (Gly-Thr-Ala-Gly-Leu-Ile-Gly-Gln). The MMP-2 sequence was added to allow controlled degradation of the hydrogel over 21 days to release the encapsulated exosomes. With the hydrogel delivery system, they showed a significantly better functional cardiac recovery, reduced scarring and lower inflammatory response 28 days after the onset of myocardial infarction, when compared to the rats treated with non-encapsulated exosomes.

## Future Directions and Conclusions

The prospect of successful tissue regeneration utilizing cell-free material, such as stem cell-derived exosomes, is exciting. The cell-free nature of exosome application circumvents the primary concern of potential tumorigenesis and unwanted mutagenesis of stem cell therapies (Cairns, 2002; Erdo et al., 2003; Volarevic et al., 2018). Delivery of therapeutic dosage of exosomes to the target site, however, has been a challenge due to their short half-life in circulation. Systemic injection of exosomes has been shown to result in rapid clearance from the blood circulation, and accumulation of the exosomes in the liver, spleen, lung, and gastrointestinal tract can be found as early as 2 h after injection (Takahashi et al., 2013; Smyth et al., 2015). The half-life of topically applied exosomes, such as on the buccal mucosa or ocular surface, may even be shorter due to the rapid fluid turnover (saliva or tears), and exposure to external elements. Hence, biodegradable or highly porous hydrogels can be utilized to incorporate exosomes in their matrices, to deliver a sustained therapeutic effect. The hydrogels can prevent the loaded exosomes from being cleared prematurely, and allow the delivery of a more localized and concentrated dosage, by placing the exosome-loaded hydrogel directly at or in the proximity of the target site. This advantage can be achieved by loading only a relatively small amount of exosomes in the hydrogels. This contrasts the potential need to deliver a repeatedly large amount of exosomes to compensate for the poor bioavailability of systemic injection.

Research into techniques to encapsulate stem cell-derived exosomes in hydrogels is still in its early stage. All of the existing methods described in the literature use polymers of natural origin, such as hyaluronic acid, chitosan, and gelatin, as the main component of the hydrogels. This is due to the fact that natural origin-based hydrogels are relatively simple to fabricate, are biodegradable or highly porous, possess similarities to native ECM, and have excellent biocompatibility. However, the potential application of hydrogel-forming synthetic polymers, such as polylactic acid (PLA) and PLGA, should not be ruled out. These synthetic polymers have been used commercially in various pharmaceutical products (Zhong et al., 2018). When considering the material characteristic of synthetic polymers, polymers that are not water-soluble, such as pHEMA, may not be suitable to encapsulate exosomes. The processing of these polymers normally involves a strong organic solvent, which may degrade the structural integrity and content of the exosomes when mixed.

Other challenges include the potential toxicity of residual unreacted cross-linkers for hydrogel making, especially for injectable hydrogels, which are designed to polymerize within the tissue. Clogging of needles may occur during injection of pH- or temperature-sensitive hydrogels. Hence, it is necessary to optimize the gelling temperature, polymer concentration, and applicator system in order to prevent premature gelation in the syringe. There is also a persistent challenge in determining the kinetic release profiles *in vivo*. The release profile generated *in vitro* often does not translate *in vivo*. The development of hydrogel-based delivery systems with a delivery rate that could be modulated on-off would be beneficial for clinical applications that require varying doses of exosomes over a period of time.

The degradability and shape of hydrogels are highly tunable. The hydrogels can also be tailored to polymerize in situ. These characteristics would allow a customizable application of an exosome delivery system. For certain clinical applications at target sites that are difficult to access, such as for the treatment of myocardial infarction, one would prefer an injectable delivery system that is able to deliver the exosomes for a longer period of time. There is a future in the commercialization of exosome-loaded hydrogel products due to their potential for patient-specific applications.

## Author Contributions

AR, HO, and GY wrote the manuscript. AR and JM conceptualized the manuscript. HO and JM obtained the grants for the study.

## Funding

This study was supported by SERI-Lee Foundation Pilot Grant (LF0618-6) awarded to HO and NMRC-funded Clinician Scientist Award-Senior Category (MOH-000197-00) awarded to JM.

## Conflict of Interest

The authors declare that the research was conducted in the absence of any commercial or financial relationships that could be construed as a potential conflict of interest.
